# Lesbian, Gay, Bisexual, and Transgender Veterans' Experiences of Discrimination in Health Care and Their Relation to Health Outcomes: A Pilot Study Examining the Moderating Role of Provider Communication

**DOI:** 10.1089/heq.2019.0069

**Published:** 2019-09-26

**Authors:** Mollie A. Ruben, Nicholas A. Livingston, Danielle S. Berke, Alexis R. Matza, Jillian C. Shipherd

**Affiliations:** ^1^ Department of Psychology, University of Maine, Orono, Maine.; ^2^ Center for Healthcare Organization and Implementation Research, VA Boston Healthcare System, Boston, Massachusetts.; ^3^ National Center for PTSD, Behavioral Science Division, VA Boston Healthcare System, Boston, Massachusetts.; ^4^ Department of Psychiatry, Boston University School of Medicine, Boston, Massachusetts.; ^5^ Department of Psychology, Hunter College of the City University of New York, New York.; ^6^ The Graduate Center of the City University of New York, New York.; ^7^ LGBT Health Program, Veterans Health Administration, Washington, District of Columbia.; ^8^ National Center for PTSD, Women's Health Sciences Division, VA Boston Healthcare System, Boston, Massachusetts.

**Keywords:** LGBT, veteran, discrimination, health care, patient–provider communication, sexual and gender minorities

## Abstract

**Purpose:** Lesbian, gay, bisexual, and transgender (LGBT) veterans report discrimination in health care, which may be associated with negative health outcomes/behaviors and has implications for LGBT identity disclosure to providers. Quality provider communication may serve to offset some of the deleterious effects of discrimination; however, no research to date has examined provider communication with respect to health among LGBT patients.

**Methods:** Participants were 47 LGBT veterans who completed measures related to past health care experiences, experiences of discrimination in health care, perceptions of provider communication, and measures of anxiety, depression, post-traumatic stress disorder symptoms, and alcohol/tobacco use.

**Results:** The majority of LGBT veterans reported experiencing LGBT-based discrimination in health care, which was associated with higher rates of tobacco use and less comfort in disclosing their LGBT identity to providers. We also found evidence of moderation, such that high-quality provider communication appeared to buffer these associations.

**Conclusion:** LGBT veterans face unique challenges with respect to receiving appropriate health care. The high frequencies of reported discrimination in health care is problematic and warrants further research and intervention. These results highlight the important role of provider communication, and the potential for quality communication to buffer against certain effects, particularly with respect to tobacco use and LGBT identity disclosure, which is an important protective factor.

## Introduction

Lesbian, gay, bisexual, and transgender (LGBT) individuals experience poorer mental and physical health relative to their heterosexual and cisgender counterparts.^1^ In part, these health disparities have been attributed to repeated exposure to stress, stigma, and discrimination both at the interpersonal level and at the institutional level (e.g., policies that allow discrimination).

A growing body of literature also documents health disparities among LGBT veterans relative to other veterans and LGBT nonveterans.^2–5^ However, much less is known about LGBT veterans' experiences in health care and associations with health behaviors.^6^ Structural and interpersonal risk factors, particularly experiencing discrimination in health care settings, may lead patients to delay treatment and/or conceal their sexual and gender identities, impacting the quality of care they receive and ultimately their health.^7^ Furthermore, modifiable health behaviors, such as smoking and substance use, might be exacerbated by structural stigma during health care visits. However, this literature has not directly examined the impact of perceived discrimination on health behaviors and health outcomes among LGBT veterans.

For LGBT patients in general, discrimination in health care is associated with poorer mental health^8^ and both lower nonmental health care utilization^8–12^ and greater mental health care utilization.^8^ Discrimination experiences in health care settings are also likely associated with more negative perceptions of health care and service providers and lower rates of disclosure about identity to health care providers.^13^ Nondisclosure, in turn, is a risk factor as it has been linked to lack of appropriate health care screenings, missed opportunities for health education by providers, and poorer patient-reported health and wellbeing.^14^ To our knowledge, discrimination in health care and its association with behavioral risk factors, such as alcohol and tobacco use, and health outcomes have yet to be explored among LGBT veteran patients. This omission is surprising, given research linking discrimination experiences generally to higher rates of alcohol and tobacco use^15,16^; we would expect similar associations in health care.

While there is limited research on the frequency and effects of discrimination among LGBT veterans, research suggests they do experience discrimination while seeking health care. In one study, veterans reported being fearful of discussing their identities with Veterans Health Administration (VHA) providers.^17,18^ In another study of lesbian veterans seeking care at VHA facilities, 10% reported experiencing harassment, 10% reported that they had been refused treatment, and 50% feared that if their VHA providers knew about their sexual orientation, they would be mistreated.^19^ In an investigation of the health care experiences of transgender veterans, participants reported experiencing insensitivity, harassment, and violence while seeking care and that VHA providers lacked knowledge about transgender care.^20^ Unfortunately, no known studies exist regarding the effects of discrimination on health behaviors and overall health among LGBT veterans.

In addition to discrimination while seeking health care, patient perceptions of provider communication also has implications for future health care seeking, adherence, and mental and physical health outcomes.^21–23^ For example, research in civilian and veteran clinical samples suggests that patients with negative perceptions of their health care and their service providers are less likely to engage in treatment and experience worse physical and mental health outcomes.^24,25^ In addition, building trusting relationships with health care providers can act as important social supports and buffer for LGBT patients. As the largest integrated health care system in the country, it is likely that the VHA is the single largest provider of health care to LGBT individuals. Therefore, understanding the role of discrimination on veteran's health care experiences, perceptions of health care quality, and health outcomes is of critical importance.

While the association between general discrimination and overall health in LGBT populations has been examined, this pilot study is the first to examine discrimination in the health care context and its association to health among LGBT veterans. In this pilot study, we document LGBT-based discrimination experiences in health care and its associations with mental health symptoms, health behaviors, and perceptions of provider communication. We hypothesized that discrimination in health care would be associated with worse mental health and health behavior outcomes, and that high-quality provider communication would buffer the negative impact of discrimination on these outcomes.

## Methods

Data from the present study comes from a mixed methods examination of LGBT veterans' trauma treatment and recovery.^26^ The study procedures were approved by Boston VA's Institutional Review Board. Participants were recruited from the local community through printed flyers, online and hardcopy advertisements. A total of 70 veterans contacted the research team and expressed interest in participating in the study. Veterans were eligible if they identified as LGBT, and endorsed experiencing at least one traumatic event that they perceived to be related to their LGBT identity. Of those who expressed interest, 11 were ineligible, 10 did not respond to follow-up calls, and 2 no-showed and did not respond to follow-up calls. The analytic sample included the 47 LGBT veterans interviewed between July 2015 and September 2016.

### Participants

Participants' ages ranged from 33 to 73 (mean=56.89, standard deviation [SD]=9.68). Participants' length of military service ranged from 1 to 28 years (mean=5.20, SD=6.95), spanning 1961 to 2016. Thirty-two identified as male, nine female, five as transgender male-to-female, and one as transgender female-to-male. Twenty-eight identified as lesbian or gay, 10 as bisexual, and 9 reported “other” as their sexual orientation. The majority of the sample was white/Caucasian (70%, *n*=33), followed by black or African American (26%, *n*=12), American Indian/Alaskan Native (2%, *n*=1); one participant did not disclose their race (Table 1).

**Table 1.** Characteristics of Lesbian, Gay, Bisexual, and Transgender Veterans (*n*=43)

Characteristics	*n* (%)
Age, years	
31–40	1 (2)
41–50	9 (21)
51–60	18 (42)
61+	15 (35)
Gender identity	
Male	28 (65)
Female	9 (21)
Male-to-female	5 (12)
Genderqueer	1 (2)
Sexual orientation	
Lesbian or gay	22 (51)
Bisexual	9 (21)
Other	12 (28)
Race	
White/Caucasian	29 (67)
Black or African American	12 (28)
American Indian/Alaska Native	1 (2)
Missing	1 (2)
Hispanic or Latino background	
Yes	6 (14)
No	37 (86)

### Materials

#### LGBT-based discrimination

The validated, reliable, and widely used Discrimination in Medical Settings Scale^27,28^ was used to examine experiences of discrimination in VHA health care. This scale has been used with racial minority samples.^10,29^ Our nine-item version asked participants to rate how frequently they had experienced discrimination (e.g., “You were called names or insulted while receiving treatment because of your LGBT identity”) while seeking health care at VHA because of their LGBT identity from “never” (0) to “almost all of the time” (5). Total scores were calculated by summing all items with higher scores indicating greater perceptions of perceived LGBT-based discrimination (Cronbach's α=0.95).

#### Perception of care

To assess perceptions of VHA physicians' interpersonal and communication skills, participants completed the 15-item Communication Assessment Tool.^30^ Participants rated different dimensions of the communication and interpersonal skills of physicians (e.g., “The healthcare provider showed care and concern”) using a 5-point rating scale from “poor” (1) to “excellent” (5; Cronbach's α=0.99). Mean scores were computed, with higher scores indicating higher quality communication.

#### Health behaviors

We used the three-item Alcohol Use Disorders Identification Test-Core (AUDIT-C)^31^ to assess the amount and frequency of drinking. The AUDIT-C is a reliable tool that focuses on the frequency of drinking, quantity consumed on a typical occasion, and the frequency of heavy episodic drinking. Scores range from 0 to 12 with higher scores representing higher alcohol consumption.

The Alcohol, Smoking, and Substance Involvement Screening Test^32^ V3.0 developed by the World Health Organization for tobacco was used. Scores ranged from 0 to 32 with higher scores representing more problematic tobacco use.

#### Mental health symptoms

Post-traumatic stress disorder (PTSD) symptomology was assessed with the PTSD Checklist (PCL-5)^33^ with Criterion A assessment. The PCL-5 is the most widely used self-report for PTSD. Participants rated the degree to which they had experienced symptoms in the last month on a scale from “not at all” (0) to “extremely” (4). An example item includes, “In the past month, how much were you bothered by repeated, disturbing dreams of the stressful experience?” (Cronbach's α=0.97). A sum total symptom severity score (range: 0–80) was calculated.

The Depression, Anxiety, Stress Scale^34^ was used to measure the three related negative emotional states of depression, anxiety, and tension/stress. The Depression scale assesses dysphoria, hopelessness, devaluation of life, self-deprecation, lack of interest/involvement, anhedonia, and inertia. The Anxiety scale assesses autonomic arousal, skeletal muscle effects, situational anxiety, and subjective experience of anxious affect. The Stress scale is sensitive to levels of chronic nonspecific arousal. It assesses difficulty relaxing, nervous arousal, and being easily upset/agitated, irritable/overreactive, and impatient. Participants rated each state over the past week on a “did not apply to me at all” (0) to “applied to me most of the time” (3) scale. Scores for Depression (Cronbach's α=0.95), Anxiety (Cronbach's α=0.91), and Stress (Cronbach's α=0.92) were calculated by summing the scores for the relevant items.

#### Disclosure to health care providers

Participants were also asked two questions about sexual orientation and/or gender identity disclosure on a scale from “not appropriate at all” (0) or “never happened to me” (1) to “very appropriate” or “happened to me all the time” (5). These questions were: “With how many of your VHA providers have you chosen to disclose your sexual orientation (and separately gender identity)?” and “How comfortable do you feel talking with your VHA providers about your sexual orientation (and separately gender identity)?”

### Demographic Information

Demographic items included year of birth, sexual orientation identity, gender identity, race, and ethnicity. Included with these questions were items related to veteran status, including dates of service.

### Analysis

Participant characteristics were summarized using mean±SD for continuous and ordinal variables and proportions for categorical variables. Associations among the study variables were examined using Pearson correlations. We examined the moderating effect of provider communication on experiences of discrimination in health care and our outcomes of interest—health behaviors, mental health, and disclosure to health care providers—using a series of multiple regressions. Moderation effects were assessed with PROCESS, a freely available computational tool for SPSS and SAS.^35^ This method calculates the bootstrapped confidence intervals using 5000 bootstrap samples. All assumptions of regression and moderation were met such that data were linear, normal, and error variances were homogenous.

## Results

Of the 47 participants, 4 were excluded because they had never sought care at VHA. Fifteen participants (32%) had never experienced discrimination while seeking care at VHA, whereas 28 participants (60%) had experienced discrimination at least once while seeking care at VHA. Table 2 summarizes the frequency of each LGBT-based discrimination experience. For zero-order correlations between study variables, see Table 3. More LGBT-based discrimination experiences in health care were related to poorer provider communication, less comfort disclosing to health care providers, and more anxiety symptoms. Higher quality perceptions of provider communication were related to greater frequency and comfort disclosing sexual orientation to health care providers, and less tobacco use. PTSD, depression, anxiety, and stress symptoms were all positively related to each other and to more alcohol use. Tobacco use was positively related to anxiety symptoms. Participants were moderately likely to disclose their sexual orientation (mean=2.90, SD=1.43) or gender identity (mean=3.50, SD=1.38) to health care providers. In addition, participants were moderately comfortable disclosing their sexual orientation (mean=3.41, SD=1.46) or gender identity (mean=3.50, SD=1.76) to health care providers.

**Table 2.** Endorsement of Items on the Discrimination in Medical Settings Scale

DMSS item	Never	Once in a while	Sometimes	A lot	Most of the time	Almost all of the time
You were treated with less courtesy than other people	25	8	5	3	0	2
You were treated with less respect than other people	20	10	7	2	1	3
You received poorer service than other people	28	6	2	4	2	1
A doctor or nurse acted as if they thought you were not smart	26	4	5	1	4	2
A doctor or nurse acted as if they were afraid of you	29	6	2	3	1	2
A doctor or nurse acted as if they thought you were dishonest	27	8	3	1	2	1
A doctor or nurse acted as if they were better than you are	24	9	3	1	4	2
You felt like a doctor or nurse was not listening to what you were saying	20	8	8	2	2	3
You were called names or insulted while receiving treatment	34	4	2	2	0	1
You were threatened or harassed while receiving treatment	34	5	1	0	3	0

*Note:* Cronbach's α=0.95.

DMSS, Discrimination in Medical Settings Scale.

**Table 3.** Correlation Matrix Showing Relationships Between Perceptions of Provider Communication, Mental Health Symptoms, Health Behaviors, and Disclosure to Health Behaviors

		1	2	3	4	5	6	7	8	9	10
1.	Discrimination in health care		−0.77^***^	−0.19	−0.38^*^	0.20	0.17	0.34^*^	0.23	0.25	0.15
2.	Perceptions of provider communication			0.34^*^	0.42^*^	−0.11	−0.09	−0.23	−0.13	−0.33^*^	−0.12
3.	Frequency of SO disclosure to HCPs				0.62^***^	−0.02	0.10	0.09	0.18	0.11	−0.06
4.	Comfort in disclosing SO to HCPs					−0.13	−0.07	−0.01	0.10	0.00	−0.02
5.	PTSD symptoms						0.84^***^	0.75^***^	0.80^***^	0.21	0.43^**^
6.	Depressive symptoms							0.83^***^	0.87^***^	0.18	0.41^**^
7.	Anxiety symptoms								0.82^***^	0.47^**^	0.41^**^
8.	Stress symptoms									0.18	0.43^**^
9.	Tobacco use										0.25
10.	Alcohol use										

*Note:* ^*^p<0.05, ^**^p<0.01, ^***^p<0.001.

HCPs, health care providers; PTSD, post-traumatic stress disorder; SO, sexual orientation.

**FIG. 1.**
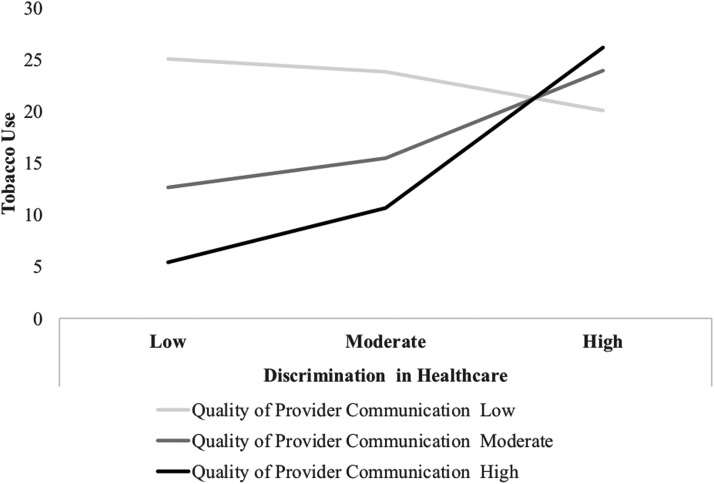
Association between experiences of discrimination in health care and tobacco use by quality of provider communication among LGBT veterans. LGBT, lesbian, gay, bisexual, and transgender.

Next, we evaluated the interaction of discrimination in health care and perceived provider communication, over and above main effects of these variables, on dependent variables of interest (Table 4). With respect to tobacco use, we found evidence of a statistically significant interaction (*b*=0.26, *p*=0.02). As displayed in Figure 1, at lower levels of discrimination, poorer quality provider communication is associated with higher rates of tobacco use and higher quality communication with less tobacco use. However, for individuals reporting higher levels of discrimination in health care, the buffering effect of provider communication is diminished. Simple slopes analyses (Table 5) suggest that the association between discrimination and tobacco use is positive and statistically significant among those reporting high levels of quality provider communication.

**Table 4.** Regression Models Testing Interactions of Experiences of Discrimination in Health Care and Perceived Quality of Provider Communication on Health Behaviors, Mental Health, and Sexual Orientation Disclosure

Predictor model	Outcome			Perceived provider communication			Interaction		
	***β***	SE	*p*	***β***	SE	*p*	***β***	SE	*p*
Health behaviors									
Tobacco use	0.28	0.21	0.19	−6.37	2.62	0.02	0.26	0.11	0.02
Alcohol use	0.05	0.06	0.38	0.13	0.17	0.44	−0.00	0.01	0.55
Mental health									
PTSD symptoms	0.43	0.49	0.39	1.60	5.94	0.79	0.01	0.25	0.97
Depressive symptoms	0.09	0.13	0.48	0.51	1.30	0.70	−0.01	0.06	0.88
Stress symptoms	0.11	0.11	0.31	0.55	1.12	0.63	−0.01	0.06	0.92
Anxiety symptoms	0.21	0.11	0.07	0.18	1.13	0.88	0.06	0.06	0.27
Sexual orientation disclosure to healthcare providers									
Frequency of disclosure to healthcare providers	0.04	0.03	0.17	0.49	0.32	0.13	0.03	0.02	0.08
Comfort in disclosing to healthcare providers	0.01	0.03	0.74	0.22	0.29	0.45	0.03	0.01	0.05

*Note*: *β* is unstandardized. *n*=36.

**Table 5.** Simple Slopes of Significant Interaction Effects

Outcome	***β***	SE	*p*
Tobacco use			
Low-quality provider communication	−0.16	0.19	0.42
Moderate-quality provider communication	0.36	0.23	0.13
High-quality provider communication	0.65	0.32	0.05
Comfort in sexual orientation disclosure to healthcare providers			
Low-quality provider communication	−0.04	0.02	0.12
Moderate-quality provider communication	0.02	0.03	0.59
High-quality provider communication	0.04	0.04	0.30

*Note*: Simple slope values taken at the 16th, 50th, and 84th percentiles. *n*=36.

**FIG. 2.**
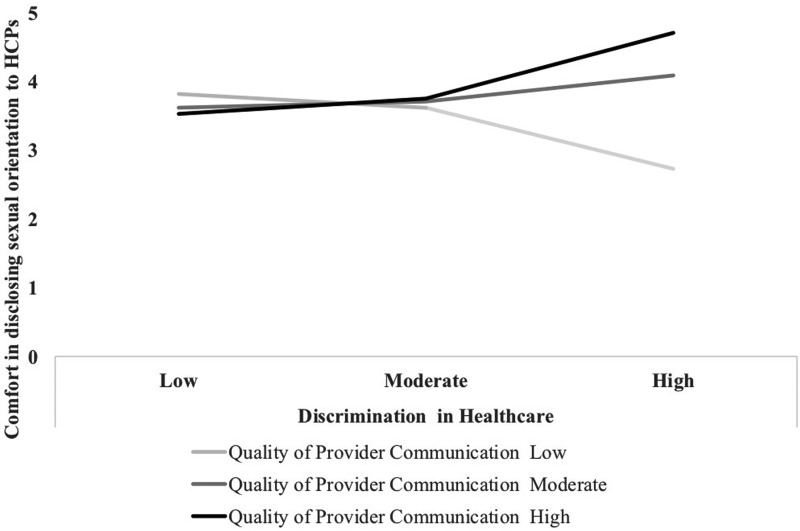
Association between experiences of discrimination in health care and comfort in disclosing sexual orientation to HCPs by quality of provider communication among LGBT veterans. HCPs, health care providers.

Only marginally significant interaction effects were found for frequency of and comfort with sexual orientation disclosure (*b_frequency_*=0.03, *p*=0.08; *b_comfort_*=0.03, *p*=0.05; Table 4). As displayed in Figure 2, at low levels of discrimination, comfort with sexual orientation disclosure was similar across levels of provider communication. At high levels of LGBT-based discrimination experiences in health care, individuals who reported poorer provider communication also reported lower comfort with sexual orientation disclosure, whereas those who reported better provider communication reported the highest degree of comfort with sexual orientation disclosure. Gender identity disclosure was not examined due to small sample size (*n*=6). No significant interactions emerged for alcohol use, anxiety, stress, depression, or PTSD symptoms.

## Discussion

This is the first known study to examine associations among LGBT-based discrimination experiences in health care, quality of care, and health-related outcomes among an LGBT veteran sample. Results showed that a majority of our sample (60%) had experienced LGBT-based discrimination at some point while seeking health care at VHA facilities. This is higher than what is reported in civilian studies,^8^ but could be related to the inclusion criteria of having experienced discrimination to participate. We hypothesized that provider communication would moderate the association between discrimination experiences in health care on health behaviors, mental health symptoms, and identity disclosure. Consistent with this hypothesis, we found a marginal interaction effect such that veterans who reported high levels of LGBT-based discrimination in health care were more comfortable disclosing their identities in the context of high-quality provider communication. Thus, it appears high-quality provider communication might buffer against disclosure apprehension in the context of previous discrimination in health care settings.

A significant interaction effect also emerged for tobacco use. For veterans reporting lower levels of LGBT-based discrimination experiences in health care, poorer provider communication was related to more tobacco use while higher quality provider communication was related to less tobacco use. However, veterans reporting higher levels of discrimination in health care endorsed similar (high) levels of tobacco use, regardless of the perceived quality of provider communication. While provider communication may buffer against low amounts of discrimination in health care, at a certain point discrimination effects may overshadow the benefits of quality provider communication. It is therefore crucial that providers are trained and aware of their communication style with their patients, and that the environment in which patients are seeking health care is welcoming and inclusive of LGBT veterans. Contrary to hypotheses, perceptions of provider communication did not moderate the relationship between discrimination experiences and alcohol use, anxiety, stress, depression, or PTSD symptoms.

Theoretical and empirical data support the potential for high-quality communication in health care settings to mitigate the negative effects of discrimination of health behavior and related outcomes. Given the strong positive correlation between perceptions of provider communication and discrimination experiences (*r*=0.77), the emergence of significant interaction effects is surprising. Both higher levels of discrimination and poorer provider communication independently predicted more tobacco use and lower frequency of and comfort in disclosing sexual orientation. However, it was the interaction of discrimination experiences and perceived provider communication that accounted for variability in tobacco use and identity disclosure in this sample.

It is possible that experiencing discrimination in health care causes patients to be more vigilant to cues displayed by providers in their verbal and nonverbal behavior—perceiving more neutral behaviors as negative.^36^ That is, even the most well-intentioned and sensitive providers may be perceived by their patients more negatively because of patients' past or current discrimination experiences in health care. This study was cross-sectional, but future research should examine how discrimination experiences impact perceptions of provider communication over time.

## Health Equity Implications

Results of the current study have important clinical implications, as tobacco use and associated health-related disparities (e.g., lung cancer)^37^ are higher among the LGBT population compared with their cisgender and heterosexual counterparts.^38^ One reason may be because LGBT individuals experience more stressful everyday experiences, including negative experiences in health care settings. Past research has suggested that providers' prejudicial attitudes are barriers to health promotion because they impede health care access.^39^ Patients may not only miss opportunities for education about risky health behaviors such as tobacco use, but irregular access to health care may increase the odds of these health risk behaviors.^40,41^ Educating providers about LGBT health disparities and potential causes (including their own behavior) could be an effective and powerful intervention to increase quality of care.^42^ Moreover, system-level interventions that are designed to reduce or eliminate disparities (e.g., LGBT-inclusive signage, inclusive name and pronoun use, and bystander interventions with anti-LGBT comments) are essential to optimizing health. In sum, health care takes place in a system, not just in an exam room, and the effects of the whole experience are relevant. It is important for future work to also examine how the intersection of identities (e.g., race, ethnicity) and cohort differences may impact the relationships as a consequence of multiple minority statuses or years of structural discrimination.

This study did not document when experiences of LGBT-based discrimination in health care occurred but regular support staff and provider training on LGBT and cultural sensitivity issues is important. Currently, there are trainings available for all VHA staff and providers, however, they are not mandated. Within VHA, providers can reassure veterans that discrimination of patients based on sexual or gender minority status is prohibited, and violations will not be tolerated within the VHA.^43,44^ Staff and providers can be trained on LGBT relevant topics, as well as provider communication and providing patient-centered care more broadly. Nonverbal behavior or even environmental factors (such as photographs on the wall) may influence veterans' health care experiences and comfort in seeking care.

In conclusion, little research exists on the experiences of LGBT patients seeking health care, and this is the first known study documenting the associations between discrimination within health care settings, provider communication, and tobacco use among LGBT veterans. Although the current data were collected as part of a pilot study, a larger scale replication is needed. High rates of discrimination within health care were noted, but could be due to inclusion criteria or experiences in health care before inclusive policies being in place. We also found that high-quality provider communication may buffer against the effects of low levels of discrimination within health care, with respect to tobacco use. However, more severe discrimination may require additional intervention beyond quality provider communication. Quality provider communication is especially important with respect to promoting identity disclosure, especially at higher levels of discrimination. From these findings, it is clear that more work is needed to reduce and overcome discrimination of LGBT veterans within health care settings. In the meantime, facilitating quality provider communication remains a priority, as it may play an important role in facilitating identity disclosure and reducing the likelihood of tobacco use among LGBT veterans.

## Abbreviations Used

AUDIT-C: Alcohol Use Disorders Identification Test-Core
DMSS: Discrimination in Medical Settings Scale
HCPs: health care providers
LGBT: lesbian, gay, bisexual, and transgender
PCL-5: PTSD Checklist
PTSD: post-traumatic stress disorder
SD: standard deviation
SO: sexual orientation
VHA: Veterans Health Administration
